# Supplementary material for: Neoadjuvant camrelizumab plus nab-paclitaxel and epirubicin in early triple-negative breast cancer: a single-arm phase II trial
Source: Nat Commun. 2023 Oct 20;14:6654. doi: 10.1038/s41467-023-42479-w (PMC10589334; doi:10.1038/s41467-023-42479-w)
Supplement: Supplementary file 1 — Supplementary Information [file 41467_2023_42479_MOESM1_ESM.pdf]

Supplementary Table 1 Pathological response in patients prematurely discontinued.

No.	Reasons for discontinuation	Total number of camrelizumab (cycles)	pCR
07	Pulmonary infection	5	Yes
09	Hyperthyroidism	1	No
12	Myelosuppression, septic shock, hypothyroidism, adrenal insufficiency	5	Yes
17	Immune-related pneumonia	5	Yes
21	Pancreatitis	4	Yes
22	Local progression	4	No
26	Immune-related pneumonia	5	No
35	Immune-related hepatic insufficiency, adrenal insufficiency	5	Yes

SUPPLEMENTARY NOTE

Neoadjuvant camrelizumab plus nab-paclitaxel and epirubicin for early triple-negative breast cancer: A single-arm phase II trial

Chengzheng Wang[1], Zhenzhen Liu[1*], Xiuchun Chen[1], Jianghua Qiao[1], Zhenduo Lu[1], Lianfang Li[1], Xianfu Sun[1], Chongjian Zhang[1], Xiayu Yue[1], Qingxin Xia[2], He Zhang[2], Min Yan[1]

Corresponding author:

Zhenzhen Liu

zlyyliuzhenzhen0800@zzu.edu.cn

Neoadjuvant camrelizumab plus nab-paclitaxel and epirubicin for early triple-negative breast cancer: A single-arm, open-label phase II trial

Study protocol

Protocol number: HR-TNBC-HN100

Version number: 2.0

Version date: May 8, 2020

Research center: Henan Cancer Hospital

Principal Investigator: Chengzheng Wang MD, PhD

Catalogue

1. Background.......13

1.1 Introduction to the research background of triple-negative breast cancer (TNBC).13

1.2 Introduction of Camrelizumab 14

1.3 Clinical Research Progress of Camrelizumab in Breast Cancer.......15

1.4 Basis of project establishment.......15

2. Objectives and endpoints.......17

2.1 Objectives.......17

2.2 Primary endpoints.......17

2.3 Secondary endpoints 18

2.4 Clinical outcomes 18

3. Research design.......19

4. Subject selection.......20

4.1 Estimation of sample size.......20

4.2 Selection Criteria.......20

***4.2.1 Inclusion Criteria*.......20**

***4.2.2 Exclusion Criteria*.......22**

***4.2.3 Dropout/Rejection Criteria* 24**

***4.2.4 Termination criteria* 24**

5. Treatment 25

5.1 Treatment Strategy 25

5.2 Dose adjustment 26

5.2.1 Administration suspension and dose adjustment of Camrelizumab....................26

5.2.2 Dose adjustment of nab-Paclitaxel..30

6. Research procedures and data collection..31

6.1 Data Collection..31

6.2 Screening and Visiting..31

6.3 Visit of treatment period...32

6.4 Visit at the end of treatment...33

6.5 Follow-up visit..34

7. Combined use of drugs..34

7.1 Drugs prohibited during the study...34

7.2 Drugs used cautiously during the study...34

7.3 Drugs and treatments that can be combined during study.......................................35

8. Evaluation of safety...36

8.1 Observation of adverse event...36

8.2 Classification of adverse event..36

8.3 Record of adverse event...36

8.4 Judgement of the relationship between an adverse event and experimental drugs..37

8.5 SAE..37

9. Data Management..39

10. Data analysis..39

10.1 Target population...39

10.2 Variables...40

10.2.1 Main Standards **..........40**

10.2.2 Other Standards **..........40**

10.3 Statistical Analysis..........40

10.3.1 Primary Analysis **..........40**

10.3.2 Secondary Analysis **..........41**

11. Research management..........41

11.1 Ethical Principles..........41

11.2 Informed consent form..........41

11.3 Laws and Regulations..........41

11.4 Confidentiality Agreement..........42

11.5 Record keeping..........42

12. Quality control and quality assurance..........42

Reference..........43

Protocol outline

**Study Title**	Neoadjuvant camrelizumab plus nab-paclitaxel and epirubicin for early triple-negative breast cancer: A single-arm, open-label phase II trial
**Version/Date**	V2.0/ May 8, 2020
**Protocol number**	HR-TNBC-HN100
**Research center**	Henan Cancer Hospital
**Research nature**	Exploratory research
**Subjects**	Patients with triple-negative breast cancer
**Study drugs**	Camrelizumab<br>Nab-paclitaxel<br>Epirubicin
**Purpose**	To evaluate the efficacy and safety of camrelizumab combined with nab-paclitaxel and epirubicin in TNBC
**Study design**	Single-arm, open-label phase II trial
**Planned enrollment**	39
**Principal investigator**	Chengzheng Wang MD, PhD

**Inclusion criteria**	1. Initially treated female patients aged ≥ 18 years.<br><br>2. ECOG score: 0-1.<br><br>3. Pathologically confirmed TNBC by core biopsy of breast tumor lesions.<br><br>Note: Negative ER and PR will be defined as ≤ 10% of cells expressing hormone receptors by IHC (immunohistochemistry) analysis. HER2 (human epidermal growth factor receptor 2) negativity will be defined as any of the following: in situ hybridization (ISH) non-amplification (ratio ≤ 2.0), or IHC 0 or IHC 1+.<br><br>4. Within 21d prior to enrollment, measurable tumor lesions will be evaluated through ultrasound or magnetic resonance imaging (MRI) in patients with tumors ≥ 2 cm in size or those 1-2 cm in size with lymph node metastasis.<br><br>5. Main organs function well.<br><br>(1) Blood routine test results: ANC≥1.5×109/L; PLT≥90×109/L; Hb≥90 g/L.<br><br>(2) Biochemical test results: TBIL ≤ upper limit of the normal value (ULN); ALT and AST ≤ 1.5 times the upper limit of the normal value (ULN); Alkaline phosphatase ≤ 2.5 times the upper limit of the normal value (ULN); BUN and Cr ≤ 1.5 × ULN and creatinine clearance ≥ 50 mL/min (CockcroftGault formula).<br><br>(3) Coagulation test results: International standardized ratio (INR) or prothrombin time (PT) ≤ 1.5×ULN, activated partial thromboplastin

	time (aPTT) ≤ 1.5×ULN.<br><br>(4) Cardiac Color Doppler and Echocardiography: Left Ventricular Ejection Fraction (LVEF≥55%).<br><br>(5) QT interval (QTcF) corrected by the Fridericia method for 18 lead electrocardiograms in females < 470ms.<br><br>6. No evidence of distant metastasis, including bilateral mammograms, breast ultrasound, chest X-ray (or chest CT scan), liver ultrasound (or liver CT scan or liver MRI), and bone scan.<br><br>7. Subjects who will have received core biopsies from tumor lesions for the determination of TNBC status and biomarker analysis before treatment begins.<br><br>8. Female patients who have not undergone menopause or surgical sterilization but have consented to observe abstinence or use effective contraceptive methods during treatment and at least 7 months after the last treatment.<br><br>9. Patients who will have signed informed consent.
**Exclusion criteria**	1. Inflammatory breast cancer or stage IV (metastatic) breast cancer.<br><br>2. Patients who have previously been treated with anti-PD-1, anti-PD-L1, anti-PD-L2 drugs or drugs targeting another co-inhibitory T cell receptor (e.g., CTLA-4, OX-40, CD137).<br><br>3. Patients with a history of anti-tumor therapy or radiotherapy for any malignant tumor, except for cured malignant tumors such as cervical carcinoma in situ, basal cell carcinoma, or squamous cell

	carcinoma.<br><br>4. Patients who are simultaneously receiving anti-tumor therapy, including endocrine therapy, bisphosphate therapy, and immunotherapy, in other clinical trials.<br><br>5. Patients who underwent major surgical procedures unrelated to breast cancer within four weeks before enrollment or those who have not fully recovered from such procedures.<br><br>6. Subjects with severe heart disease or discomfort, including but not limited to the following diseases:<br><br>-- Heart failure or systolic dysfunction (LVEF<50%);<br><br>-- High-risk uncontrolled arrhythmias, such as atrial tachycardia, resting heart rate>100 bpm, significant ventricular arrhythmias (e.g., ventricular tachycardia), or a higher-level atrioventricular block (i.e., Mobitz II second degree atrioventricular block or third-degree atrioventricular block);<br><br>-- Angina requiring treatment with anti-angina drugs;<br><br>-- Clinically significant valvular heart disease;<br><br>-- Transmural myocardial infarction indicated by ECG;<br><br>-- Poor control of hypertension (systolic blood pressure>180 mmHg and/or diastolic blood pressure>100 mmHg);<br><br>7. Patients suffering from autoimmune diseases or other diseases requiring systemic treatment with corticosteroids or immunosuppressive drugs (physiological corticosteroid replacement

	therapy that allows adrenal or pituitary dysfunction).<br><br>8. Those with primary or acquired immunodeficiency diseases (e.g., allogeneic organ transplantation).<br><br>9. Pregnant and lactating women, those with fertility and positive baseline pregnancy test results, or women of reproductive age who are unwilling to take effective contraception measures during the trial and within 7 months after administering the last dose of study medication.<br><br>10. Patients with a history of (non-communicable) pneumonia or currently have pneumonia that requires steroid treatment.<br><br>11. Women with an active or previously documented inflammatory bowel disease (e.g., Crohn's disease, ulcerative colitis).<br><br>12. Patients with a known history of the following infections;<br><br>-- Human immunodeficiency virus (HIV)<br><br>-- Acute or chronic hepatitis B or C<br><br>-- The live virus vaccine was administered within 30 d of the planned start date of treatment. The study allows seasonal influenza vaccines that do not contain live viruses.<br><br>13. Patients with a known history of allergy to the drug components in this protocol; those with a history of immunodeficiency, including a positive test for HIV or other acquired congenital immunodeficiency diseases, or a history of organ transplantation.<br><br>14. Patients suffering from severe or other concomitant diseases that

	may interfere with the planned treatment or any other condition in which the investigator believes that the patient is ineligible for participation.
**Treatment strategy**	Camrelizumab (200 mg) will be administered intravenously on day 1 every three weeks (no less than 30 min and no more than 60 min) for 6 consecutive cycles.<br><br>Nab-Paclitaxel (125 mg/m^2) will be administered intravenously on days 1, 8, and 15 every three weeks for 6 consecutive cycles.<br><br>Epirubicin (75 mg/m^2) will be administered intravenously on day 1 every three weeks for 6 consecutive cycles.<br><br>Participants who either discontinued or completed the neoadjuvant therapy could undergo surgery as clinically indicated, and their pathological tumor response will be evaluated. After surgery, clinicians will conduct postoperative chemotherapy or radiotherapy for patients in accordance with the clinical treatment guidelines and institutional standard procedures.<br><br>Subsequent treatment will be administered to subjects whose medication has been discontinued due to toxicity and those who do not qualify for surgery, in accordance with the institutional standard procedures. Safety assessment will be conducted 28 d after the last administration of the study medication. The study will monitor disease recurrence and disease-free survival of subjects through a follow-up of at least 5 years.<br><br>The above drugs can be dosed according to the protocol based on particpants' adverse responses. The study will allow for the down-

	regulation of chemotherapy drugs up to two times, and their final dose should not be less than 75% of the total dose. Patients will continue to receive treatment until the disease progresses, toxicity becomes intolerable, withdrawal is informed, or the investigator determines that medication must be stopped. The administration cycle for participants will be determined from the date of the first administration. In the event of missed medication or suspension of treatment due to adverse events, investigators will need to record in detail, the time at which the drug should have been taken and the reasons for not taking it, and then continue with treatment according to the protocol cycle without making supplemental or weekly adjustments.
**Estimation of sample size**	Five patients will be selected to evaluate safety, and if the results are satisfactory, a more extensive efficacy study will be conducted. Notably, existing literature and clinical experience show that the pCR rate of anthracycline combined with taxanes in triple-negative breast cancer neoadjuvant chemotherapy is about 30%. It is estimated that the pCR rate of preoperative treatment using camrelizumab combined with nab-paclitaxel and epirubicin can reach 55%. Therefore, using Simon's two-stage method with an α value of 5%, and test efficiency of 80%, the estimated sample size will be 35 cases. The initial phase of the study will require 9 patients to be enrolled. Out of these, 5 patients have already undergone safety assessment, and the remaining 4 will need to be enrolled again. In addition, at least 4 out of the 9 patients must reach pCR (pathological complete response) in order to advance to the next stage of the study. In the second stage, 26 patients will be enrolled, and if 15 or more reach pCR, the trial will be considered successful. Considering a 10%

	patient dropout rate, a total of 39 patients will need to be enrolled.
**Study endpoints**	**Primary endpoints:**<br>● Pathological complete response (pCR) rate<br>**Secondary endpoints**<br>● Safety<br>● Event-free survival (EFS)<br>● Disease-free survival (DFS)<br>● Distant disease-free survival (DDFS)<br>● Objective remission rate (ORR) evaluated using the Response Evaluation Criteria in Solid Tumors (RECIST1.1)

1. Background

1.1 Introduction to the research background of triple-negative breast cancer (TNBC)

Breast cancer is the most commonly diagnosed cancer and the leading cause of cancer deaths among Chinese women [1]. Moreover, the age of cancer onset in China is lesser than that in Western countries. It is estimated that there were 1.67 million newly diagnosed cases of breast cancer worldwide in 2012. In China alone, for instance, the number of newly diagnosed breast cancer cases each year is estimated to be around 160000, among which 120000 patients die of the disease. The general classification of breast cancer is based on the expression of estrogen receptor (ER), progesterone receptor (PgR), and human epidermal growth factor receptor 2 (HER2). However, based on the gene expression study, Perou and Sorlie et al. proposed five molecular subtypes of breast cancer with unique clinical manifestations, viz., Luminal A, Luminal B, Her-2 enrich, and normal-like and basal-like breast cancers [2] [3]. TNBC is the most common breast cancer that accounts for approximately 70% of all breast cancer cases; among TNBC, the basal-like subtype constitutes 70-80% of the total [4].

TNBC, predominantly observed in young women, is a highly invasive breast cancer that, accounts for 10–20% of all breast cancers. It is characterized by the lack of expression of ER, PgR, and HER-2 receptors. Patients with TNBC often suffer from advanced diseases, have a high incidence of metastasis and recurrence, and have a significantly poor prognosis. Targeted therapy has little effect on TNBC. Furthermore, the tumor can quickly become resistant to chemotherapy, and the median overall survival (OS) time is only 8–13 months. When compared with hormone receptor-positive (HR+) tumors, TNBC has poor differentiation, higher proliferation rate, higher recurrence rate, higher incidence of distant metastasis, and overall significantly poor survival rate [5]. In the past 20 years, the OS rate of patients with TNBC has not improved, which highlights the need to enrich the treatment options for these patients [6]. Unlike TNBC, several targeted therapies can benefit patients with other breast cancer subtypes like hormone receptor-positive and HER2-positive breast cancers. However, as per the Chinese Clinical Oncology Association (CSCO), sequential chemotherapy remains the standard

treatment for TNBC patients in both preoperative-neoadjuvant and postoperative-adjuvant settings. In patients with this type of breast cancer, the response rate of standard chemotherapy is low (10–15%) and the progression-free survival time is very short (2-3 months) [7]. In addition, most patients have suffered disease progression after receiving first-line treatment, and the choice of treatment remains limited to chemotherapy.

1.2 Introduction of camrelizumab

Immune blockade inhibitors, also known as immune checkpoint inhibitors anti-(programmed cell death 1 [PD-1] or anti-(programmed cell death ligand 1 [PD-L1] inhibitor, ICI), are a new type of monoclonal antibody drugs that can inhibit the function of inhibitory immune receptors in order to evoke an immune antineoplastic response. PD-1 is a recently discovered immune checkpoint that protects against autoimmunity through two mechanisms. First, it promotes apoptosis (programmed cell death) of antigen-specific T-cells in lymph nodes. Second, it reduces apoptosis in regulatory T-cells (anti-inflammatory, suppressive T cells). This prevents autoimmune diseases, but it can also prevent the immune system from killing cancer cells. PD-1 has two ligands PD-L1 and PD-L2. Several reports suggest that PD-1 along with its ligands negatively regulates immune response. Overexpression of PD-L1 in tumor cells inhibits anti-tumor activity through engagement with PD-1 molecules on T-lymphocytes. This engagement transmits negative regulatory signals, which lead to the apoptosis and immune energy of tumor antigen-specific T-cells, ultimately facilitating tumor cells escape immune monitoring and killing.

PD-1 inhibitors have become a new class of tumor immunotherapy drugs, which regulate the anti-tumor activity of T-lymphocytes and cause tumor apoptosis by blocking the PD-1/PD-L1 signal pathway. Camrelizumab (SHR-1210), a humanized monoclonal antibody against PD-1, is one such immune-blockade inhibitor recently developed in China by Jiangsu Hengrui Pharmaceutical Co., Ltd. Preclinical research data demonstrated that SHR-1210 has considerable *in vivo* efficacy and safety when compared with similar drugs abroad.

1.3 Clinical Research Progress of Camrelizumab in Breast Cancer

The phase I/II clinical trials of SHR-1210 on multiple tumor types were conducted by Jiangsu Hengrui Pharmaceuticals Co., Ltd. in Australia and China simultaneously from 2015. These trials preliminarily verified the safety, tolerance, and efficacy of the Carrell monoclonal antibody (SHR-1210) in the treatment of advanced solid tumors. The phase II clinical study regarding the efficacy and safety of SHR-1210 in combination with apatinib mesylate in patients with advanced TNBC showed good efficacy. As of January 30, 2019, the ORR reached 47.4%, the DCR reached 68.4%, and the mPFS has not yet been achieved. The only common adverse reactions reported in this study were fatigue, hand and foot syndrome, and elevated aspartate aminotransferase/glutamic pyruvic transaminase (AST/ALT). Importantly, no treatment-related death was reported. In general, SHR-1210 combined with apatinib mesylate has significant efficacy and controllable safety in the treatment of advanced TNBC. In another study, the safety and efficacy of SHR-1210 in combination with apatinib mesylate and fluzopalil in the treatment of TNBC were explored. The study included patients with advanced metastatic or recurrent TNBC and at least failed first-line chemotherapy. The end point of the study was dose-limited toxicity (DLT).

In addition, a comprehensive phase Ib/II study led by Professor Zhimin Shao of the Affiliated Cancer Hospital of Fudan University was designed to evaluate the efficacy and safety of multiple targeted treatments in patients with refractory and metastatic TNBC. This study included 140 patients with TNBC, who were divided into 7 groups and were treated with different targeting and chemotherapeutic drugs, respectively. The patients were followed up on the progression of the disease, with ORR as the main endpoint.

1.4 Basis of project establishment

Neoadjuvant therapy is the standard treatment for locally advanced breast cancer. It has long been used for its impact on surgery, downstaging tumors, and allowing breast-conserving surgery rather than mastectomy. Neoadjuvant therapy tests new therapeutic and predictive

biomarkers by providing tumor and blood samples before and during systemic treatment, besides allowing rapid evaluation of drug effectiveness. It can also reduce axillary staging to avoid axillary dissection and require only sentinel lymph node biopsies [8] [9] [10]. Pathological complete response (pCR) of neoadjuvant therapy is associated with disease-free survival (DFS) and OS of early breast cancer. The correlation between pathological reaction and long-term survival of patients with early breast cancer was the strongest among TNBC patients, followed by HER2-positive patients, and the lowest correlation was detected in hormone receptor-positive patients [8] [11].

KEYNOTE-173 is an international multi-cohort phase Ib, study which evaluated six chemotherapy regimens in combination with pembrolizumab as the preoperative neoadjuvant treatment for TNBC patients [12]. The main inclusion criteria were women older than or equal to 18 years old; previously untreated, locally advanced TNBC; ECOG score 0–1; better liver and kidney function. Women with fertility potential needed to take adequate contraceptive measures during the study. The main exclusion criteria were metastatic breast cancer with bilateral invasive breast cancer or inflammatory breast cancer; another malignant tumor in the past 5 years, excluding basal cell carcinoma of the skin, squamous cell carcinoma of the skin, or cervical cancer *in situ*. Received chemotherapy, targeted therapy, radiotherapy, immunotherapy for immune checkpoints, co-stimulation or co-inhibition of T cell receptors, and active autoimmune diseases within 12 months before enrollment. Systemic treatment is required in the past 2 years; diagnosed with immunodeficiency or receiving systemic steroid therapy or any other form of immunosuppressive therapy within 7 days before the first dose of the study drug; with a known history of the human immunodeficiency virus (HIV). The primary end point was DLTs, and the secondary end points included pathological complete response rate (pCR) and objective response rate (ORR).

A total of 60 patients with TNBC were randomly assigned into 6 cohorts; each cohort was combined with different doses of carboplatin, combined with pembrolizumab, and sequential doxorubicin and cyclophosphamide. The specific drug administration regimens of each cohort were as follows: cohort A: 125 mg/m^2 i.v. d1,8,15 q3w; pembrolizumab 200 mg i.v. d1 q3w;

cohort B: nab-paclitaxel 100 mg/m^2 i.v. d1,8,15 q3w; carboplatin AUC 6 d1 q3w, pembrolizumab 200 mg i.v. d1 q3w. cohort C: nab-paclitaxel 125 mg/m^2 i.v. d1,8,15 q3w; carboplatin AUC 5 d1 q3w, pembrolizumab 200 mg i.v. d1 Q3w; cohort D: nab-paclitaxel 125 mg/m^2 i.v. d1,8,15 q3w; pembrolizumab 200 mg i.v. d1 q3w; carboplatin AUC 2 d1,8,15 q3w; pembrolizumab 200 mg i.v. d1 q3w; cohort E: paclitaxel 80 mg/m^2 i.v. d1,8,15 q3w; carboplatin AUC 5 d1 q3w; pembrolizumab 200 mg i.v. d1 q3w; cohort F: paclitaxel 80 mg/m^2 i.v. d1,8,15 q3w; carboplatin AUC 2 d1 q3w; pembrolizumab 200 mg i.v. d1 q3w. Then, all cohorts were sequenced with pembrolizumab 200 mg i.v. d1 q3w, doxorubicin 60 mg/m^2 i.v. d1 q3w, and cyclophosphamide 600 mg/m^2 i.v. d1 q3w. The results suggested that the pCR rate (ypT0/Tis ypN0) of the overall population is 60% (90% CI, 49% CI 71%), and the pCR rate of each queue ranges from 30% to 80%. Thus, it can be seen that PD-1 inhibitors combined with chemotherapy can achieve better efficacy in the neoadjuvant therapy of TNBC.

In clinical practice, the total number of cycles of neoadjuvant chemotherapy plus adjuvant chemotherapy is 6–8 cycles. Chemotherapy is generally not considered if all adjuvant chemotherapy cycles have been completed during neoadjuvant chemotherapy.

Here, a single-arm, prospective, open clinical study was conducted to use camrelizumab combined with nab-paclitaxel and epirubicin in the preoperative treatment of TNBC, including 39 patients with TNBC. This study further confirmed the efficacy and safety of camrelizumab combined with nab-paclitaxel and epirubicin in the treatment of TNBC.

2. Objectives and endpoints

2.1 Objectives

To evaluate efficacy and safety of preoperative treatment of triple-negative breast cancer using camrelizumab combined with nab-paclitaxel and epirubicin.

2.2 The primary endpoints

- Pathological complete response (pCR) rate

2.3 Secondary endpoints

- Safety
- Event-free survival (EFS)
- Disease-free survival (DFS)
- Distant disease-free survival (DDFS)
- Objective remission rate (ORR) evaluated using the Response Evaluation Criteria in Solid Tumors (RECIST1.1)

2.4 Clinical outcomes

Primary efficacy end point:

tpCR (ypT0/is, ypN0), evaluated by pathologists from the research center, will be defined as the absence of residual invasive carcinoma (by pathologic assessment) in H&E stained, resected breast cancer samples and all ipsilateral lymph node samples following neoadjuvant treatment and surgery.

Secondary efficacy end points:

Event-free survival (EFS) was defined as the time from enrollment to the first recorded relevant event, where the relevant events include preoperative disease progression, postoperative disease recurrence, and death from any cause.

Disease-free survival (DFS) was defined as the time from the first disease-free day (i.e., the date of surgery) to the first recorded relevant event, where the relevant events include postoperative disease recurrence and death from any cause.

Distant disease-free survival (DDFS) was defined as the time from the first disease-free day (i.e., the date of surgery) to the first recorded distant disease.

Objective remission rate (ORR) will be defined as the subject ratio for optimized tumor

relief when CR or PR is reached during the neoadjuvant treatment. In this study, ORR was evaluated according to the Response Evaluation Criteria in Solid Tumors (RECIST v1.1).

3. Research design

This will be a single-arm, prospective, open-label clinical study that aims to explore the safety and efficacy of preoperative treatment of triple-negative breast cancer using camrelizumab combined with nab-paclitaxel and epirubicin.

First, five subjects who meet the inclusion criteria will be recruited for safety assessment by receiving treatment with the trial drug (camrelizumab for injection) combined with nab-paclitaxel and epirubicin. Camrelizumab and epirubicin will be administered intravenously on the first day of each treatment course, with a cycle of 21d. nab-Paclitaxel will be administered intravenously on the 1st, 8th, and 15th days of each treatment course, with a cycle of 21d. The cycle duration will be calculated based on the start and end time of the administration cycle of camrelizumab.

Subjects who meet the enrollment criteria will be selected for the second stage of the extended efficacy study, with their safety also being evaluated, provided that their safety is deemed satisfactory, any Level 3 adverse responses are manageable, and there are no adverse responses categorized as Level 4-5. The treatment strategy will be similar to the one above, and they will be continuously accepted for 6 cycles. Upon completion of 6 cycles of neoadjuvant treatment, all subjects who qualify for surgery will receive surgical treatment, after which their pathological remissions will be evaluated. The patients will receive treatment until we observe; objective disease progression, worsening of symptoms, unacceptable toxicity, death, or withdrawal of consent, regardless of which of these occurs first.

Clinicians will administer postoperative chemotherapy or radiotherapy to subjects with clinical indications after surgery, according to the clinical treatment guidelines and treatment protocols of the respective research centers.

In this study, a follow-up of at least five years will be implemented to monitor disease

recurrence and DFS. Efficacy will be evaluated after completing treatment/terminating follow-up for all subjects. Patient safety during the entire duration of the study will be evaluated through laboratory tests and adverse-event reports.

4. Subject selection

4.1 Estimation of sample size

Initially, 5 patients will be selected to evaluate the safety, and if the results are satisfactory, a more extensive efficacy study will be conducted. Notably, existing literature and clinical experience show that the pCR rate of anthracycline combined with taxanes in triple-negative breast cancer neoadjuvant chemotherapy is about 30%. It is estimated that the pCR rate of preoperative treatment using camrelizumab combined with nab-paclitaxel and epirubicin can reach 55%. Therefore, using Simon's two-stage method with an α value of 5%, and test efficiency of 80%, the estimated sample size will be 35 cases. The initial phase of the study will require 9 patients to be enrolled. Out of these, 5 patients will have already undergone safety assessment, and the remaining 4 will need to be enrolled again. In addition, at least 4 out of the 9 patients must reach pCR (pathological complete response) in order to advance to the next stage of the study. In the second stage, 26 patients will be enrolled, and if 15 or more reach pCR, the trial will be considered successful. Considering a 10% patient dropout rate, a total of 39 patients will need to be enrolled.

A total of 39 cases will be enrolled in this study.

4.2 Selection criteria

4.2.1 Inclusion criteria

1. Initially treated female patients aged ≥ 18 years.

2. ECOG score: 0-1.

3. Pathologically confirmed TNBC by core biopsy of breast tumor lesions.

Note: Negative ER and PR will be defined as ≤ 10% of cells expressing hormone receptors by IHC (immunohistochemistry) analysis. HER2 (human epidermal growth factor receptor 2) negativity will be defined as any of the following: *in situ* hybridization (ISH) non-amplification (ratio ≤ 2.0), or IHC 0 or IHC 1+.

4. Within 21d prior to enrollment, measurable tumor lesions will be evaluated through ultrasound or magnetic resonance imaging (MRI) in patients with tumors ≥ 2 cm in size or those 1-2 cm in size with lymph node metastasis.

5. Main organs function well.

(1) Blood routine test results: ANC≥1.5×10^9/L; PLT≥90×10^9/L; Hb≥90 g/L.

(2) Biochemical test results: TBIL ≤ upper limit of the normal value (ULN); ALT and AST ≤ 1.5 times the upper limit of the normal value (ULN); Alkaline phosphatase ≤ 2.5 times the upper limit of the normal value (ULN); BUN and Cr ≤ 1.5 × ULN and creatinine clearance ≥ 50 mL/min (CockcroftGault formula).

(3) Coagulation test results: International standardized ratio (INR) or prothrombin time (PT) ≤ 1.5×ULN, activated partial thromboplastin time (aPTT) ≤ 1.5×ULN.

(4) Cardiac color doppler and echocardiography: Left Ventricular Ejection Fraction (LVEF≥55%).

(5) QT interval (QTcF) corrected by the Fridericia method for 18 lead electrocardiograms in females < 470ms.

6. No evidence of distant metastasis, including bilateral mammograms, breast ultrasound, chest X-ray (or chest CT scan), liver ultrasound (or liver CT scan or liver MRI), and bone scan.

7. Subjects who will have received core biopsies from tumor lesions for the determination of TNBC status and biomarker analysis before treatment begins.

8. Female patients who have not undergone menopause or surgical sterilization but have consented to observe abstinence or use effective contraceptive methods during treatment and at least 7 months after the last treatment.

9. Patients who will have signed informed consent.

4.2.2 Exclusion criteria

1. Inflammatory breast cancer or stage IV (metastatic) breast cancer.

2. Patients who have previously been treated with anti-PD-1, anti-PD-L1, anti-PD-L2 drugs or drugs targeting another co-inhibitory T cell receptor (e.g., CTLA-4, OX-40, CD137).

3. Patients with a history of anti-tumor therapy or radiotherapy for any malignant tumor, except for cured malignant tumors such as cervical carcinoma *in situ*, basal cell carcinoma, or squamous cell carcinoma.

4. Patients who are simultaneously receiving anti-tumor therapy, including endocrine therapy, bisphosphate therapy, and immunotherapy, in other clinical trials.

5. Patients who underwent major surgical procedures unrelated to breast cancer within four weeks before enrollment or those who have not fully recovered from such surgical procedures.

6. Subjects with severe heart disease or discomfort, including but not limited to the following diseases:

-- Heart failure or systolic dysfunction (LVEF<50%);

-- High-risk uncontrolled arrhythmias, such as atrial tachycardia, resting heart rate>100 bpm, significant ventricular arrhythmias (e.g., ventricular tachycardia), or a higher-level atrioventricular block (i.e., Mobitz II second degree atrioventricular block or third-degree atrioventricular block);

-- angina requiring treatment with anti-angina drugs;

-- clinically significant valvular heart disease;

-- transmural myocardial infarction indicated by ECG;

-- poor control of hypertension (systolic blood pressure>180 mmHg and/or diastolic blood pressure>100 mmHg);

7. Patients suffering from autoimmune diseases or other diseases requiring systemic treatment with corticosteroids or immunosuppressive drugs (physiological corticosteroid replacement therapy that allows adrenal or pituitary dysfunction).

8. Those with primary or acquired immunodeficiency diseases (e.g., allogeneic organ transplantation).

9. Pregnant and lactating women, those with fertility and positive baseline pregnancy test results, or women of reproductive age who are unwilling to take effective contraception measures during the entire duration of the trial and within 7 months after the administration of the last study medication.

10. Patients with a history of (non-communicable) pneumonia or currently have pneumonia that requires steroid treatment.

11. Women with an active or previously documented inflammatory bowel disease (e.g., Crohn's disease, ulcerative colitis).

12. Patients with a known history of the following infections;

-- Human immunodeficiency virus (HIV)

-- acute or chronic hepatitis B or C

-- the live virus vaccine was administered within 30 d of the planned start date of treatment.

The study will allow the use of seasonal influenza vaccines that do not contain live viruses.

13. Persons with a known history of allergy to the drug components in this protocol; those with a history of immunodeficiency, including a positive test for HIV, or other acquired, congenital immunodeficiency diseases, or a history of organ transplantation.

14. Patients suffering from severe or other concomitant diseases that may interfere with the planned treatment or any other condition in which the investigator believes that the patient is unsuitable for participation in this study.

4.2.3 Dropout/Rejection Criteria

1. Inability to assess efficacy and/or safety due to failure of medication to comply with the provisions of this protocol.

2. Severe protocol violation; for instance, when the participant receives treatment with other anti-tumor drugs during the study.

4.2.4 Termination criteria

1. When a participant withdraws his/her informed consent and requests to leave the study.

2. When pregnancy events occur during the study.

3. When the subject is unable to tolerate toxicity.

4. When disease progression occurs.

5. Other instances where the researcher believes that the subject needs to withdraw from the study.

5. Treatment

5.1 Treatment strategy

Camrelizumab (200 mg) will be administered intravenously, every three weeks (no less than 30 min and no more than 60 min), in 6 consecutive cycles, with a cycle consisting of 21d. Injectable Camrelizumab is produced by Jiangsu Hengrui Pharmaceutical Co., Ltd.

Nab-Paclitaxel (125 mg/m^2) will be administered intravenously on days 1, 8, and 15, in a 21-d cycle, for 6 consecutive cycles. Injectable Paclitaxel (albumin-binding) is produced by Jiangsu Hengrui Pharmaceutical Co., Ltd.

Epirubicin (75 mg/m^2) will be administered intravenously on day 1, in a 21-d cycle, for 6 consecutive cycles.

Participants who discontinue or complete the neoadjuvant therapy could undergo surgery, and their pathological remission will be evaluated. Pathological evaluation of tumor efficacy will be conducted by the research center before and after surgery. After surgery, clinicians will conduct postoperative chemotherapy or radiotherapy for patients in accordance with the clinical treatment guidelines and institutional standard procedures.

Subsequent treatment will be administered to subjects whose medication has been discontinued due to toxicity and those who do not qualify for surgery, in accordance with the institutional standard procedures. Safety assessment will be conducted 28 d after the last administration of the study medication. The study will monitor disease recurrence and disease-free survival of subjects through a follow-up of at least 5 years.

The above drugs can be dosed according to the protocol, based on participants' adverse responses. The study will allow for the downregulation of chemotherapy drugs up to two times, and their final dose should not be less than 75% of the total dose. Patients will continue to receive treatment until the disease progresses, toxicity becomes intolerable, withdrawal is informed, or the investigator determines that medication must be stopped. The administration

cycle for participants will be determined from the date of the first administration. In the event of missed medication or suspension of treatment due to adverse events, investigators will need to record in detail, the time at which the drug should have been taken and the reasons for not taking it, and then continue with treatment according to the protocol cycle without making supplemental or weekly adjustments.

5.2 Dose adjustment

5.2.1 Administration suspension and dose adjustment of Camrelizumab

(1) PD-1 monoclonal antibody administration delay standard:

●Any ≥ Level 2 non-skin, medicine-related adverse events, except for Level 2 medicine-related fatigue do not require delayed treatment.

●Level 2 medicine-related creatinine clearance is increased.

●Any medicine-related skin AE ≥ 3.

●Any abnormalities ≥ Level 3 with medicine-related laboratory tests, excluding the following regarding lymphopenia, abnormalities of AST, ALT, or total bilirubin, and asymptomatic amylase or lipase.

-Level 3 lymphopenia does not require delayed administration.

- If the baseline level of subjects' AST, ALT, or total bilirubin is within the normal range, delayed administration occurs when ≥ Level 2 medicine-related toxicity occurs.

- If the baseline level of subjects' AST, ALT, or total bilirubin is within the level 1 toxicity range, delayed administration occurs when ≥ Level 3 medicine-related toxicity occurs.

- Any drug-related amylase or lipase abnormalities ≥ Level 3 that are not related to the symptoms or clinical manifestations of pancreatitis do not require delayed administration.

●Any adverse events, laboratory abnormalities, or concurrent diseases that the investigator determines require delayed administration of the study drug. It is recommended to monitor every three days until AST/ALT reaches the highest value and begins to decrease.

(2) Recovery PD-1 monoclonal antibody medication standards:

If the response to medicine-related AE reaches ≤ 1 level or baseline, subjects can resume treatment with the study drug, with the following exceptions:

●Subjects can restart treatment if they have level 2 fatigue.

●Subjects with no Level 3 medicine-related skin AE can restart treatment if they have Level 2 skin toxicity.

●For subjects with elevated AST, ALT, or total bilirubin at Level 2, treatment can be resumed after laboratory values have returned to the baseline level and glucocorticoid therapy (if necessary) is completed.

●Subjects with a combination of AST/ALT and total bilirubin Level 2 elevated values that meet the withdrawal criteria should be permanently discontinued.

●Medicine-related pulmonary toxicity, diarrhea, or colitis must be alleviated to baseline level before starting treatment again.

●Subjects with persistent Level 1 pneumonia can be re-treated at least one month after the end of steroid dose reduction.

●Subjects with medicine-related endocrine diseases < Level 4 can be fully controlled by using physiological hormone replacement therapy and can be re-treated.

(3) Standard for permanent discontinuation of PD-1 monoclonal antibody:

●Any Level 2 drug-related uveitis, eye pain, blurred vision, ineffective local treatment,

and failure to recover to ≤ Level 1 after delayed medication; Or the above AE requiring systemic treatment.

●Any Level 3 medicine-related non-skin AE with a duration >7 d, with the following exceptions:

-- Any Level 3 drug-related uveitis, pneumonia, bronchospasm, hypersensitivity reactions, or infusion reactions must be terminated from the study and treatment.

--Level 3 drug-related endocrine diseases, only hormone replacement therapy with physiologic dose is required and fully controllable, and there is no need to terminate treatment.

-- Level 3 abnormal drug-related laboratory tests do not require termination of treatment, but if Level 3 thrombocytopenia occurs for >7 d or is related to bleeding, the study medication must be terminated.

●Comply with the following hepatotoxicity results:

--AST/ALT>10 times of ULN for two weeks.

--AST/ALT>15 times of ULN.

--When at baseline, subjects with elevated total bilirubin appear >8 times of ULN, while those with normal total bilirubin appear >5 times of ULN.

--When at baseline, subjects with normal total bilirubin appear simultaneously with AST/ALT >3 times of ULN and total bilirubin >5 times of ULN. When at baseline, subjects with elevated total bilirubin appear simultaneously with AST/ALT >3 times of ULN and total bilirubin>8 times of ULN.

●Any Level 4 drug-related AE or laboratory test abnormality, except for the following:

--Level 4 granulocytopenia <7 d.

--Level 4 lymphopenia is decreased or leukopenia is reduced.

-- Isolated Level 4 amylase or lipase elevation is not accompanied by pancreatitis symptoms or clinical manifestations. The sponsor needs to be informed of the occurrence of Level 4 amylase or lipase elevation.

--Isolated Level 4 electrolyte imbalance/abnormality that is not accompanied by clinical sequelae and can be corrected by supplementation/appropriate treatment within 72 hours of its occurrence.

--Level 4 medicine-related endocrine disease only requires hormone replacement therapy with physiological doses which is fully controllable and does not require termination of treatment.

●Need >6 weeks of medication delay, must terminate PD-1 monoclonal antibody treatment, except for the following:

-- Delayed administration of the cortisol hormone for>6 weeks due to the need to gradually reduce the dose will be allowed when dealing with drug-related adverse events. A decision must be made after discussion with the sponsor before resuming administration. During the delayed administration period, tumor evaluation should be continued according to the provisions of the protocol. Safety follow-up and laboratory tests should also be conducted at the original frequency or more frequently when there are clinical indications.

-- If treatment needs to be delayed for more than 6 weeks due to non-study drug-related reasons, a decision must be made after discussion with the sponsor before resuming administration. During the delayed treatment period, tumor evaluation should be continued according to the protocol requirements. Safety follow-up and laboratory tests should also be conducted at the original frequency or more frequently when there are clinical indications.

●Any adverse events, laboratory abnormalities, or concurrent diseases that, according to the judgment of the researcher, will pose significant risks to the participants if the study of the

drug is continued.

●Disease progression evaluated by researchers according to RECIST 1.1.

Even if the use of PD-1 monoclonal antibody is terminated, tumor evaluation must continue according to the protocol requirements

5.2.2 Dose adjustment of nab-Paclitaxel

(1) Hepatic dysfunction:

There will be no need to treat patients who have mild hepatic dysfunction with dose adjustment (1 × ULN < total bilirubin ≤ 1.5 × ULN and AST ≤ 10 × ULN). Instead, they will be treated using the same dose as that in used patients with normal liver function.

For patients who have medium and severe hepatic dysfunction (1.5×ULN < total bilirubin ≤ 5×ULN and AST≤10×ULN), it is recommended to reduce the dose by 20%. If the patient shows tolerance to two courses of treatment with lower doses, the dose can be increased to the dosage used 3

Treatment should be discontinued in patients with total bilirubin >5×ULN or AST >10×ULN.

(2) Sensory neurotoxicity:

Medication should be suspended in patients with Level 3 sensory neurotoxicity, and treatment can only continue when neurotoxicity returns to ≤ Level 2.

(3) Neutropenia:

The dosage of nab-Paclitaxel should be reduced in patients with severe neutropenia (for one week or above) during treatment. If severe neutropenia occurs again, the dosage should continue to be reduced.

6. Research procedures and data collection

6.1 Data collection

The duration of safety data collection is 30 days after the patient signed ICF to the end of the last medication. All adverse events were recorded in the CRF from the signing of the ICF to the end of the study. The severity of adverse events will be evaluated according to the NCICTCAE4.0 criteria.

Each patient will receive a planned visit and specific data will be recorded at different time points of the visit. All examinations/tests are recommended, and the specific examination/testing items shall prevail according to the clinical practice.

6.2 Screening and visiting

The following screening steps must be completed within 2 weeks before starting the study of drug treatment unless otherwise noted:

- Collect the signed informed consent forms.
- Collect patients' demographic data: gender, date of birth, nationality, height, weight, etc.
- Collect data on the vital signs: heart rate, respiratory rate, body temperature, and blood pressure.
- Collect ECOG score.
- Take medical histories and treatment histories, such as diabetes, hypertension, or chronic obstructive pulmonary disease.
- Record the diagnosis of the tumor: date of diagnosis, histological classification, location of lesion (primary or metastatic lesion), pathological stage TNM, and clinical stage.
- Record the coagulation function.
- Record the blood routine test results.

- Record the urine/stool routine (+ occult blood) results.
- Record the blood biochemistry results.
- Record the thyroid function test results.
- Record the results of pregnancy tests (women of child-bearing age).
- Record the tumor hormone receptor status and biomarkers.
- Record the estrogens.
- Record the ANA.
- Record the ACTH.
- Record the results of imaging examination: breast CT or MRI (enhanced), other external parts of the breast can be examined by imaging examination such as the head, chest, upper abdomen, and pelvic cavity.
- Record the electrocardiogram data.
- Record the echocardiography data.
- Record the efficacy of the combined use of drugs: the rational use of drugs should be recorded within 30 days before being included in the group.

6.3 Visit of the treatment period

Examination during the treatment period:

- Vital signs: heart rate, respiratory rate, body temperature, and blood pressure.
- Physical examination: head and face, skin system, lymph nodes, eyes, ear, nose and throat, oral cavity, respiratory system, cardiovascular system, abdomen, genitourinary system, musculoskeletal, nervous system, and mental state (including skin toxicity, nausea, fatigue, and tooth functions such as loosening or tooth loss).
- ECOG score.

- Coagulation function.
- Blood routine.
- Urine/stool routine (+ occult blood).
- Blood biochemistry.
- Thyroid function.
- Tumor biomarkers.
- Electrocardiogram.
- Echocardiography.
- Efficacy evaluation: CT or MRI (both enhanced) at the end of every 2 cycles, or when the researcher deemed it necessary.
- Combined use of drugs and adverse events: observed and recorded at any time.

6.4 Visit at the end of treatment

At the end of the study or withdrawal from the study, if the patient does not have an examination within 14 days before the end of the study, the following tests should be performed:

- Vital signs, physical examination, and blood pressure examination.
- Laboratory examination: blood routine, urine routine, stool routine (+occult blood), blood biochemistry, tumor biomarkers, coagulation function, and thyroid function.
- Electrocardiogram.
- Echocardiography.
- ECOG score and QoL score.
- Combined use of drugs and adverse event recording.

Imaging examination: If an imaging examination is not performed within 4 weeks before the

end of treatment, an imaging examination should be conducted at the end of the study. For patients showing progression of non-imaging evidence (intolerable, otherwise), a tumor assessment is performed every 3 months or when the researcher deems it necessary until the disease progresses, death, or starting other tumor treatment.

6.5 Follow-up visit

The patient began the post-treatment follow-up after the last use of the study drug. Patients who leave the group due to disease progression should follow up on the survival time, once every 3 months. Telephone follow-up is also acceptable.

During the follow-up period, the following parameters should be recorded: the time of disease progression or death; other tumor treatment; and the survival time of SAE (telephone follow-up is available and records need to be kept).

The study of adverse events that have not recovered at the time of drug withdrawal should be followed up and finally evaluated. All patients should be followed up for 30 days after the last medication to find any new adverse events.

7. Combined use of drugs

7.1 Drugs prohibited during the study

Other antineoplastic drugs, including proprietary Chinese patent medicines with specific antineoplastic indications.

7.2 Drugs used cautiously during the study

If there are adverse reactions, the subjects should be closely monitored and actively treated if necessary, and the drugs used should be recorded and described on the CRF table. The following drugs were used with caution during the study:

Drugs that interfere with liver P450 enzymes:

1. CYP3A4 inducers (catarimide, rifampicin, and phenobarbital) and inhibitors (ketoconazole, itraconazole, erythromycin, and clarixan);
2. Substrates of CYP3A4 (simvastatin, cyclosporine, and piperidine);
3. Other drugs metabolized by CYP3A4 (such as benzodiazepines, dihydropyridine, calcium antagonists, and HMG-COA reductase inhibitors);
4. Substrates of CYP2C9 (diclofenac, phenytoin, Piroxicam, S-warfarin, and toluenesulfonbutylurea) and substrates of CYP2C19 (diazepam, imipramine, lansoprazole, and S-mefentoine).

Drugs for prolonging the QT intervals:

1. Antibiotics (such as clarithromycin, azithromycin, erythromycin, roxithromycin, metronidazole, and moxifloxacin, etc.);
2. Antiarrhythmic drugs (quinidine, sotalol, amiodarone, propylamide, and procainamide, etc.);
3. Antipsychotic drugs (risperidone, fluphenazine, droperidol, haloperidol, thiolidazine, pimozide, olanzapine, clozapine, etc.);
4. Antifungal agents (fluconazole, ketoconazole, etc.);
5. Antimalarial drugs (mefloquine, chloroquine, etc.);
6. Antidepressants (amitriptyline, imipramine, clomipramine, doxepin, and doxepin).

7.3 Drugs and treatments that can be combined during the study

Clinical concomitant diseases and all types of AEs should be treated actively. Natural medicines used before can continue to be used during the study and recorded in the appropriate eCRF. All kinds of drugs used together should be recorded in eCRF in strict accordance with the regulations of GCP.

8. Evaluation of safety

8.1 Observation of adverse event

Definition of AE: AE refers to any adverse medical event that occurs after the subject has received a drug or treatment, but does not necessarily have a causal relationship with the treatment.

AE can be any unpleasant physical signs (including abnormal laboratory results), symptoms, or diseases that are related to the use of medical products or have nothing to do with the purpose of using the products.

According to the regulations, events that occur before and after treatment are also considered to be AE. Therefore, the report of the safety monitoring AE or SAE should start from the subject entering the trial, which includes signing the informed consent form, to the end of the trial.

8.2 Classification of adverse event

According to the “common acute and subacute toxicity grading standard” (NCI-CTCAE 5.0), AE has been divided into five grades ranging from 0 to 5 if AE is not listed in the NCI toxicity classification standard, it can be judged according to the following criteria:

Degree I (mild): there is an uncomfortable feeling, but it does not affect the routine activities.

Degree II (moderate): the uncomfortable feeling that reduces or affects routine activities.

Degree III (severe): an inability to work or perform routine activities.

Grade IV (fatal): maimed or fatal.

8.3 Record of adverse event

The names, severity, occurrence time, duration, treatment measures, and prognosis of all AEs during the trial should be recorded in detail, and truthfully filled in the case report form (CRF).

The abnormal laboratory test data are recorded on the CRF table and repeated at least once a week, followed up until normal or at the end of the study.

Adverse events within 30 days after the end of the last treatment should be reported and recorded.

8.4 Judgement of the relationship between adverse events and experimental drugs

The possible association between AE and experimental drugs is evaluated according to the five-level classification of "definitely relevant, likely relevant, possibly relevant, possibly irrelevant, and irrelevant". Among the five, the first three levels are related to the experimental drugs, where the incidence of adverse reactions is calculated by taking the three together as numerators, and the total number of subjects used to evaluate safety as the denominator.

8.5 SAE

(1) Determination of serious adverse events (SAEs)

SAE refers to medical events resulting in prolonged hospitalization, disability, affecting working ability, endangering life, and leading to congenital malformations during clinical trials. Following unexpected medical events are included in SAE:

- An event leading to death.
- A life-threatening event (defined as a subject at risk of death at the time of the event).
- Events requiring hospitalization or extension of hospitalization duration.
- Events that can lead to permanent or severe disability/dysfunction.
- Congenital abnormalities or birth defects.
- Drug overdose.

(2) Pregnancy

Pregnancy during clinical trials should be reported in accordance with SAEs.

(3) Disease progression

Disease progression (including symptoms and the signs of progression) should not be reported as SAEs. Instead, deaths resulting from disease progression within the trial or safety reporting period should be reported as SAEs. Furthermore, hospitalization due to symptoms and signs of disease progression should not be reported as an SAE.

(4) Other antineoplastic therapy

If the subjects start other antineoplastic therapy, the reporting period for non-fatal adverse events is up to the start of new antineoplastic therapy. If the death occurs within the reporting period of SAEs after the end of the study treatment, it must be reported regardless of whether the patient is receiving other treatment.

(5) Hospitalization treatment

Adverse events that lead to hospitalization or prolonged hospitalization in clinical studies should be regarded as SAEs. Any initial hospitalization by a medical institution (even if less than 24 h) meets this standard.

The following conditions do not include in hospitalization:

Rehabilitation institutions; sanatoriums; routine emergency room admission; same-day surgery (such as outpatient/same-day/non-bedridden surgery) hospitalization or extension of hospitalization that has nothing to do with the deterioration of adverse events are not SAEs.

Hospitalization for health management reasons (e.g., annual routine medical examination); hospitalization under the trial program during clinical trials (e.g., operating as required by the trial program) elective hospitalization not related to the deterioration of adverse events (e.g., elective cosmetic surgery); scheduled treatment or surgery should be recorded throughout the

trial program and/or in the subjects' baseline data; hospitalization only for the use of blood products.

Diagnostic or therapeutic invasive (such as surgery) and non-invasive procedures of a disease condition should not be reported as adverse events until meet the definition of an adverse event. For instance, acute appendicitis during the reporting period should be reported as an adverse event; therefore, appendectomy should be recorded as the treatment of the adverse event.

(6) Drug overdose

Drug overdose means that the subjects take additional drugs more than the dose prescribed by the researcher within 24 h (the specific time is adjusted according to the specific plan). All trial drug overdoses, regardless of whether they are related to adverse events/SAEs, should be reported as SAEs.

9. Data management

In this study, the preservation of research medical records will be crucial as they are the most unique records. The medical record report will be obtained from the research medical record and will be completed by the researcher. Selected cases must complete the case report form. After reviewing the completed case report form, the first copy will be submitted to the main research unit for data entry and management. After the first copy is handed over, the contents of the case report form will remain the same.

10. Data analysis

10.1 Target population

● Full Analysis Set (FAS): According to the principle of intention-to-treat analysis (ITT), all patients who will have taken the drug at least once will be included in the efficacy analysis. For case data that fail to observe the entire treatment process, the last observation data will be used to transfer to the final test result (LOCF).

● Per-Protocol Set (PPS): All cases that comply with the trial protocol, have good compliance, and have taken drugs for at least one cycle (except for those with clear medical evidence due to disease progression after enrollment), have not taken prohibited drugs during the trial, and have completed the CRF requirements. Do not fill in any missing data. The efficacy of the drug will be analyzed statistically using both FAS and PPS.

● Safety Analysis Set (SAS): All enrolled patients who will have used the trial medication at least once and have a safety record after medication will belong to SAS. This set will be used for safety analysis.

10.2 Variables

10.2.1 Main standards

pCR rate: No residual invasive carcinoma (by pathological assessment) was detected by H&E-staining of resected breast samples and all sampled lymph nodes after completion of neoadjuvant treatment.

10.2.2 Other standards

EFS, DFS, DDFS, ORR, and medicine-related safety indicators.

10.3 Statistical analysis

10.3.1 Primary analysis

① pCR rate: Ratio of postoperative pCR patients to PPS patients.

② Overall survival and progression-free survival curves were developed using the Kaplan-Meier method. In addition, median survival, median progression-free survival, and 95%CI were estimated.

10.3.2 Secondary analysis

The association, incidence rate, severity, and risk factors for important, known adverse events, and the association, incidence rate, severity, and risk factors for important potential adverse events (Level 3-4 diarrhea, gastrointestinal fistula/perforation, wound healing complications, reversible posterior leukoencephalopathy syndrome, decreased cardiac LVEF/heart failure, thrombosis/embolism, hepatotoxicity, hypothyroidism). The ORR, disease control rate, and 95% CI will be calculated. The improvement-of-the-quality-of-life score at the end of each week compared to the baseline will be calculated and tested, respectively.

11. Research management

11.1 Ethical principles

The study will be conducted in accordance with the principles established by the 18th Joint Congress of the World Medical Association (Helsinki, 1964) and all subsequent amendments.

11.2 Informed consent form

The subjects must have informed consent to participate in the trial before receiving the drug administration in order to protect the legitimate rights and interests of the subjects. The researcher has the responsibility to give a complete and comprehensive introduction to the purpose of this study, the possible side effects and possible risks of the effects of the drug to the subjects or their designated representatives, and should let the subjects know their rights, risks, and benefits. Conversation is a very important informed consent process. If the subject and his legal representative are unable to read, the informed consent process shall be attended by a witness, who shall sign the informed consent form after oral consent by the subject or his legal representative, and the witness's signature shall be taken on the same day as the subject's signature. The informed consent form shall indicate the version number and date.

11.3 Laws and Regulations

This study will be conducted in accordance with the applicable laws and regulations.

11.4 Confidentiality agreement

Without the prior formal written consent of Henan Cancer Hospital, the researcher or any member of his/her team may not disclose these materials or materials to unauthorized persons.

Except for information permitted by regulations to be disclosed, researchers shall keep confidential all information received, obtained, or derived during the course of the study and shall take all necessary steps to ensure that the information is not disclosed.

11.5 Record keeping

The researcher should arrange for the safekeeping of the research documents until the end of the study. In addition, with regard to patient record keeping, researchers should comply with specific local regulations/guidelines.

12. Quality control and quality assurance

The researchers must be doctors who were trained in clinical trials and worked under the guidance of senior professionals. The pre-trial inspection of the clinical ward must meet the standardized requirements to ensure that the rescue equipment is complete. It is suggested that the subjects should be given drugs by professional nurses for a better understanding of the use of drugs and to ensure the compliance of the subjects. The research center must strictly follow the research plan and timely input e-CRF monitors should follow the standard operating procedures, supervise the conduct of clinical trials, and confirm that all data records and reports are correct and complete.

Reference

[1]. Wanqing Chen, Rongshou Zheng. Incidence, mortality and survival analysis of breast cancer in China. Chin J Clin Oncol 2015, 42(13):668-674.

[2]. Perou CM, Sørlie T, Eisen MB, et al. Molecular portraits of human breast tumours. Nature 2000, 406, 747–752.

[3]. Sorlie T, Perou CM, Tibshirani R, et al. Gene expression patterns of breast carcinomas distinguish tumor subclasses with clinical implications. Proc. Natl. Acad. Sci. U. S. A. 2001, 98, 10869–10874.

[4]. Prat A, Adamo B, Cheang MCU, et al. Molecular Characterization of Basal-Like and Non-Basal-Like Triple-Negative Breast Cancer. Oncologist 2013, 18, 123–133.

[5]. Nedeljković M, Damjanović A. Mechanisms of Chemotherapy Resistance in Triple-Negative Breast Cancer-How We Can Rise to the Challenge. Cells. 2019 Aug 22;8(9). pii: E957.

[6]. Voorwerk L, Slagter M, Horlings HM, et al. Immune induction strategies in metastatic triple-negative breast cancer to enhance the sensitivity to PD-1 blockade: the TONIC trial. Nat Med. 2019 Jun;25(6):920-928.

[7]. Bardia A, Mayer IA, Vahdat LT, et al. Sacituzumab Govitecan-hziy in Refractory Metastatic Triple-Negative Breast Cancer. N Engl J Med. 2019 Feb 21;380(8):741-751.

[8]. Cortazar P, Zhang L, Untch M, et al. Pathological complete response and long-term clinical benefit in breast cancer: the CTNeoBC pooled analysis. Lancet. 2014;384(9938):164-172.

[9]. Killelea BK, Yang VQ, Mougalian S, et al. Neoadjuvant chemotherapy for breast cancer increases the rate of breast conservation: results from the National Cancer Database. J Am Coll Surg. 2015;220(6):1063-1069.

[10]. King TA and Morrow M. Surgical issues in patients with breast cancer receiving

neoadjuvant chemotherapy. Nat Rev Clin Oncol. 2015;12(6):335-343.

[11]. Kim MM, Allen P, Gonzalez-Angulo AM, et al. Pathologic complete response to neoadjuvant chemotherapy with trastuzumab predicts for improved survival in women with HER2-overexpressing breast cancer. Ann Oncol. 2013;24(8):1999-2004.

[12]. Schmid P, Salgado R, Park Y H, et al.Pembrolizumab plus chemotherapy as neoadjuvant treatment of high-risk, early-stage triple-negative breast cancer: results from the phase 1b open-label, multicohort KEYNOTE-173 study[J].Ann Oncol,2020, 31 (5): 569-581.
