# Supplementary material for: Neoadjuvant camrelizumab plus nab-paclitaxel and epirubicin in early triple-negative breast cancer: a single-arm phase II trial
Source: Nat Commun. 2023 Oct 20;14:6654. doi: 10.1038/s41467-023-42479-w (PMC10589334; doi:10.1038/s41467-023-42479-w)
Supplement: Supplementary file 3 — Reporting Summary [file 41467_2023_42479_MOESM3_ESM.pdf]

nature portfolio

Corresponding author(s): Zhenzhen Liu

Last updated by author(s): Sep 15, 2023

Reporting Summary

Nature Portfolio wishes to improve the reproducibility of the work that we publish. This form provides structure for consistency and transparency in reporting. For further information on Nature Portfolio policies, see our Editorial Policies and the Editorial Policy Checklist.

Please do not complete any field with "not applicable" or n/a. Refer to the help text for what text to use if an item is not relevant to your study.
For final submission: please carefully check your responses for accuracy; you will not be able to make changes later.

Statistics

For all statistical analyses, confirm that the following items are present in the figure legend, table legend, main text, or Methods section.

n/a	Confirmed	
☐	☒	The exact sample size (*n*) for each experimental group/condition, given as a discrete number and unit of measurement
☒	☐	A statement on whether measurements were taken from distinct samples or whether the same sample was measured repeatedly
☐	☒	The statistical test(s) used AND whether they are one- or two-sided<br>*Only common tests should be described solely by name; describe more complex techniques in the Methods section.*
☒	☐	A description of all covariates tested
☒	☐	A description of any assumptions or corrections, such as tests of normality and adjustment for multiple comparisons
☐	☒	A full description of the statistical parameters including central tendency (e.g. means) or other basic estimates (e.g. regression coefficient) AND variation (e.g. standard deviation) or associated estimates of uncertainty (e.g. confidence intervals)
☒	☐	For null hypothesis testing, the test statistic (e.g. *F*, *t*, *r*) with confidence intervals, effect sizes, degrees of freedom and *P* value noted<br>*Give P values as exact values whenever suitable.*
☒	☐	For Bayesian analysis, information on the choice of priors and Markov chain Monte Carlo settings
☒	☐	For hierarchical and complex designs, identification of the appropriate level for tests and full reporting of outcomes
☐	☒	Estimates of effect sizes (e.g. Cohen's *d*, Pearson's *r*), indicating how they were calculated

Our web collection on statistics for biologists contains articles on many of the points above.

Software and code

Policy information about availability of computer code

Data collection	Microsoft Excel and SPSS statistics 26 version R26.0.0.0 were used for data collection.
Data analysis	SPSS statistics 26 version R26.0.0.0, SAS version 9.4, and PASS version 15.0.3 softwares were used for data analysis and figure generation.

For manuscripts utilizing custom algorithms or software that are central to the research but not yet described in published literature, software must be made available to editors and reviewers. We strongly encourage code deposition in a community repository (e.g. GitHub). See the Nature Portfolio guidelines for submitting code & software for further information.

Data

Policy information about availability of data

All manuscripts must include a data availability statement. This statement should provide the following information, where applicable:

- Accession codes, unique identifiers, or web links for publicly available datasets
- A description of any restrictions on data availability
- For clinical datasets or third party data, please ensure that the statement adheres to our policy

The study protocol is available as Supplementary Note in the Supplementary Information file. The raw patient data are protected and not available due to the data privacy laws. The individual de-identified patient data will be made available upon reasonable request by contacting the corresponding author (zlyyliuzhenzhen0800@zzu.edu.cn). The remaining data are available within the Article, Supplementary Information or Source Data file. Source data are provided with this paper.

Human research participants

Policy information about studies involving human research participants and Sex and Gender in Research.

Reporting on sex and gender	This study reported on only one sex based on self-report and body characteristics. Only the female patients with early triple-negative breast cancer were eligible for inclusion. The results of the study were only apply to one sex. No sex analysis was therefore carried out.
Population characteristics	The study included female patients with histologically confirmed treatment-naïve non-metastatic TNBC, defined as ER and PR negative or low expression by immunohistochemistry (PR or ER staining in <10% of tumor cells irrespective of intensity) and HER2 negative by immunohistochemistry. Median age of enrolled patients was 46 years old. Patients were enrolled in the study at The Affiliated Cancer Hospital of Zhengzhou University and treated with camrelizumab and chemotherapy (nab-paclitaxel and epirubicin).
Recruitment	Patients with non-metastatic triple-negative breast cancer (TNBC) were offered for potential participation at The Affiliated Cancer Hospital of Zhengzhou University. All patients had treatment-naïve breast cancer. Patients were excluded if they had inflammatory breast cancer, previously received anti-PD-1, anti-PD-L1, anti-PD-L2 drugs or drugs targeting another co-inhibitory T cell receptor, anti-tumor therapy or radiotherapy for any malignant tumors, participated in other clinical trials, underwent major surgical procedures unrelated to breast cancer within four weeks before enrollment or those who have not fully recovered from such procedures, with severe heart disease or discomfort, suffering from autoimmune diseases or other diseases requiring systemic treatment with corticosteroids or immunosuppressive drugs, pregnancy, suffering from severe or other concomitant diseases that may interfere with the planned treatment or any other condition in which the investigator believes that the patient is ineligible for participation. no limitations according to prior lines of therapy. The broad inclusion criteria and prospective nature of the clinical trial, minimized potential bias.
Ethics oversight	This study was performed in compliance with the Good Clinical Practice guideline and Declaration of Helsinki (revised in 2013). The study protocol and any amendments were approved by Institutional Review Board of the Affiliated Cancer Hospital of Zhengzhou University (Ethics number: 2019110712). All patients provided written informed consent before participation.

Note that full information on the approval of the study protocol must also be provided in the manuscript.

Field-specific reporting

Please select the one below that is the best fit for your research. If you are not sure, read the appropriate sections before making your selection.

☒ Life sciences ☐ Behavioural & social sciences ☐ Ecological, evolutionary & environmental sciences

For a reference copy of the document with all sections, see nature.com/documents/nr-reporting-summary-flat.pdf

Life sciences study design

All studies must disclose on these points even when the disclosure is negative.

Sample size	The null hypothesis of a pCR rate of 30% was adopted for neoadjuvant concurrent anthracycline and nab-paclitaxel chemotherapy in this study. Assuming a pCR rate of 55% would be achieved following neoadjuvant camrelizumab plus anthracycline and nab-paclitaxel chemotherapy, a total of 39 patients (including nine in the first stage) were required when considering a one-sided α of 5%, a power of 80%, and a drop-out rate of 10%.
Data exclusions	All patients enrolled in this study were included for analysis.
Replication	Not applicable. This is a single-arm phase II clinical trial, involving assessment of Chemotherapy plus immune checkpoint inhibitors in human participants. No preclinical data that could be replicated is provided in the manuscript.
Randomization	Not applicable. We did not include a control arm in this study. This was a single-arm phase II trial to investigate the efficacy and safety of camrelizumab in combination with nab-paclitaxel and epirubicin chemotherapy in the neoadjuvant therapy of early TNBC. The primary endpoint was pathological complete response.
Blinding	Not applicable. This was an open label clinical trial. Given that there was no randomization, and patients were included in one consecutive cohort, the blinding did not apply.

Reporting for specific materials, systems and methods

We require information from authors about some types of materials, experimental systems and methods used in many studies. Here, indicate whether each material, system or method listed is relevant to your study. If you are not sure if a list item applies to your research, read the appropriate section before selecting a response.

Materials & experimental systems

n/a	Involved in the study	
☐	☒	Antibodies
☒	☐	Eukaryotic cell lines
☒	☐	Palaeontology and archaeology
☒	☐	Animals and other organisms
☐	☒	Clinical data
☒	☐	Dual use research of concern

Methods

n/a	Involved in the study	
☒	☐	ChIP-seq
☒	☐	Flow cytometry
☒	☐	MRI-based neuroimaging

Antibodies

Antibodies used

PD-L1 expressions were determined using the PD-L1 IHC 22C3 pharmDx kit (Dako Agilent, Dako North America, Inc., Carpinteria, CA, USA).

Validation

The PD-L1 IHC 22C3 pharmDx kit is a FDA-approved IHC companion in vitro diagnostic assay

Clinical data

Policy information about clinical studies

All manuscripts should comply with the ICMJE guidelines for publication of clinical research and a completed CONSORT checklist must be included with all submissions.

Clinical trial registration

NCT04213898

Study protocol

The full clinical trial protocol has been uploaded as supplementary note.

Data collection

Data was collected using Microsoft Excel and SPSS statistics 26 at The Affiliated Cancer Hospital of Zhengzhou University.

In terms of the recruitment period, the study was open to accrual on 1st January 2020, with the first patient enrolled on the January 3, 2020 and last patient enrolled on October 25th, 2021. The data collection started with the enrollment of the first patient on January 2020 and the follow-up is ongoing.

Outcomes

The primary endpoint was pathological complete response (ypT0/is ypN0), evaluated by pathologists from the research center, and was defined as the absence of residual invasive carcinoma (by pathologic assessment) in H&E stained, resected breast cancer samples and all ipsilateral lymph node samples following neoadjuvant treatment and surgery.
Secondary endpoints included safety, event-free survival (EFS), disease-free survival (DFS), distant disease-free survival (DDFS) , objective response rate (ORR). The ORR was evaluated according to the Response Evaluation Criteria in Solid Tumors (RECIST v1.1).

This checklist template is licensed under a Creative Commons Attribution 4.0 International License, which permits use, sharing, adaptation, distribution and reproduction in any medium or format, as long as you give appropriate credit to the original author(s) and the source, provide a link to the Creative Commons license, and indicate if changes were made. The images or other third party material in this article are included in the article's Creative Commons license, unless indicated otherwise in a credit line to the material. If material is not included in the article's Creative Commons license and your intended use is not permitted by statutory regulation or exceeds the permitted use, you will need to obtain permission directly from the copyright holder. To view a copy of this license, visit http://creativecommons.org/licenses/by/4.0/
